# Personal

**Published:** 1902-02

**Authors:** 


### PERSONAL.

Dr. Alice Barlow Brown, of Chicago, upon the request of a number of homeopathic physicians of this city, has decided to take up her residence in Buffalo and practise her profession. It is expected that Dr. Brown will succeed to the practice of the late Dr. Jessie Shepard. Office and residence: 36 Irving Place. Hours: 11 to 3 and 7 to 8 p.m.; Sunday, 3 to 4 p.m. Telephone, Tupper 292.

---

Dr. G. Frank Lydston, of Chicago, visited Buffalo January 21, 1902. In the afternoon he visited Niagara Falls in company with some Buffalo physicians, and in the evening he read a paper before the Buffalo Academy of Medicine, entitled, Relation of evolution to infection, with special reference to venereal diseases.

---

Dr. Henry L. Elsner, of Syracuse, president of the Medical Society of the State of New York, read a paper before the Medical Society of the County of Chautauqua, December 17, 1901, entitled, Arteriosclerosis in early and middle life. Dr. Elsner was elected an honorary member of the society.

---

Dr. Edwin Ricketts, of Cincinnati, president of the American Association of Obstetricians and Gynecologists, will read a paper entitled, Hematoma of the ovary, before the Brooklyn Gynecological Society at its regular meeting, February 7, 1902.

---

Dr. J. Henry Dowd, Dr. William C. Krauss and Dr. William Warren Potter, of Buffalo, were elected honorary members of the Medical Society of the County of Chautauqua at its meeting, held at Jamestown, December 17, 1901.

---

Dr. Lewis C. Bosher, of Richmond, Va., was elected president of the Richmond Academy of Medicine at its annual meeting December 10, 1901.

Dr. P. W. Van Peyma, of Buffalo, has been reappointed a member of the city school board for the term of five years. In view of Dr. Van Peyma's efficient work in the past, it is difficult to see how Mayor Knight could have done otherwise than recognise it by giving him a second term.

